# Self-Rated Health of US Older Adults in the General Social Survey (GSS) 1972–2021: Complexity of the Associations of Education and Immigration

**DOI:** 10.3390/healthcare11040463

**Published:** 2023-02-05

**Authors:** Hafifa Siddiq, Mona Darvishi, Babak Najand

**Affiliations:** 1 School of Nursing, Charles R Drew University of Medicine and Science, Los Angeles, CA 90059, USA; 2 Marginalization-Related Diminished Returns, Los Angeles, CA 90059, USA

**Keywords:** older adults, self-rated health, education, immigration, United States

## Abstract

Background: Multiple studies have shown a link between high education and better self-rated health (SRH). However, recent studies have suggested that immigrants may experience a weaker association between education and SRH than native-born individuals. Aim: Using a national sample of US older adults, this investigation studied whether there is an inverse association between education and SRH and whether immigration status moderates this association. Methods: This study is based on marginalized diminished returns (MDRs) that argues socioeconomic status (SES) resources, such as education, may generate less favorable health outcomes for marginalized groups. Data were from the General Social Survey (GSS) 1972–2021, a cross-sectional survey in the US. A total of 7999 participants who were 65+ years old were included. The independent variable was education, measured as years of schooling and treated as a continuous variable. The dependent variable was poor/fair (poor) SRH. Immigration status was the moderator. Age, sex, and race were control variables. Logistic regressions were used for data analysis. Results: We found that higher levels of education were protective against poor SRH. However, this effect was weaker for immigrants than for US-born individuals. Conclusions: This study found that native-born US older individuals are more likely to experience the protective effect of their education against poor SRH compared to their immigrants. Eliminating health inequality between immigrant and US-born individuals needs policies that go beyond socioeconomic status (SES) equality and address barriers that hinder highly-educated immigrants.

## 1. Introduction

Education is an essential socioeconomic status (SES) indicator that affects a wide range of health outcomes that are not limited to self-rated health (SRH) [1]. Research has consistently shown that people with higher education have lower chronic diseases, better mental health, and longer life expectancy [2,3]. Higher education is also associated with SRH [4].

The health-promoting effects of education [5], however, may differ between majority and minority groups [6,7,8], a phenomenon called marginalization-related diminished returns (MDRs) [9,10]. For example, among 25,659 US adults, highly educated people were protected against developing cardiac disease, however, this protective effect was weaker in ethnic minorities than non-Hispanic White people [11]. Multiple studies have documented MDRs in various settings, which can be defined as the attenuated positive impact of education on the health status of marginalized groups, compared to the majority and privileged group [12,13,14,15,16,17,18,19,20,21,22,23,24]. This is in part because people from marginalized groups have limited access to opportunity structures, such as high-paying jobs and safe neighborhoods, because of social stratification, racism, and discrimination. As such, despite high education, minority groups may take fewer advantages of their education in terms of living conditions [4,25,26,27,28]. These differential returns of education differ from lower education in minority and marginalized populations. This is important because most research has historically focused on the role of lower education (and other SES indicators) of minoritized groups; therefore, there is a need to understand factors that may contribute to sustained health disparities between majority and minority populations who have higher education levels. It is plausible that racism and discrimination, particularly through lower pay jobs, would reduce the health returns of education for minorities so they may become unhealthy even when they attained higher education.

Multiple studies have examined the role of immigration status, as a marginalizing identity, on the positive health effects of education [19,29,30,31,32]. One recent study showed that immigration status moderates the association between education and the SRH of adults in Europe [33]. Although we know that the association between education and SRH exists [34,35,36,37,38,39,40,41,42,43,44,45,46,47], there may be a weaker association between education and SRH among immigrants than their US-born counterparts [33]. Another study used data from 30 European countries and suggested that education affects SRH; however, this effect depended upon immigration status (weaker for immigrants than native-born people). That study called for more studies to evaluate the role of immigration on changing the health returns of education, as the MDRs theory suggests [48]. 

However, we are not aware of any similar studies in older adults. Some studies in older adults show that ethnic minority status may reduce the health and economic returns of educational attainment. A longitudinal study from the Alzheimer’s Disease Neuroimaging Initiative (ADNI) study followed middle-aged and older US adults with Alzheimer’s disease (AD) to explore racial differences in the association between educational attainment and memory. Among 1673 American middle-aged and older adults, education showed a significant interaction with race on memory, with higher educational attainment having a stronger effect on memory in White patients than Black patients [49]. Another analysis used data from the Health and Retirement Study (HRS 1992-ongoing), a nationally representative longitudinal study that followed 10,023 middle-aged and older adults (50+ years old) for up to 26 years. The study aimed to explore the racial variation in the predictive utility of baseline education level on protecting people against poor self-rated health (SRH), body mass index (BMI), and depressive symptoms (DS). Cluster analysis was used to categorize individuals into low and high-risk groups (outcome) based on SRH, BMI, and DS, using race as a moderator. While overall, high education level reduced the odds of poor SRH, BMI, and DS over the span of 26 years of follow up, there were statistically significant interactions between race and education on all three health outcomes. These interactions indicated that protective effects of baseline educational attainment on poor health over time are smaller for Black than White older adults [50]. All these studies suggest that MDRs are also relevant to older adults; however, all previous studies have used race as the marginalizing identity. Thus, more research is needed in older adults to test whether immigration status also alters the health returns of education or not.

### Aims

To fill this gap in the literature, we aimed to study the association between education and SRH in the US general population of older adults and moderating effects of immigration status on the association between education and poor SRH. First, we hypothesized an inverse association between education and poor SRH in older adults overall. Secondly, we hypothesized that this association would be weaker for immigrants than for US-born people.

## 2. Methods

This cross-sectional study used data from the General Social Survey (GSS) conducted from 1972 to 2021 in the US. GSS is a cross-national survey founded and continuously conducted since 1972 to study the pattern of social changes in US populations.

### 2.1. Study Population

GSS has used a random sample over all GSS years (cumulative sample). Thus, the results are generalizable to the US population of adults. Samples were selected between years of 1972 to 2021 from all US states.

### 2.2. Analytical Sample

For the purpose of this analysis, any participant with valid data on education, immigration status, and SRH was included. The total sample included in this analysis was 7999 individuals aged 65+ who lived in the US. Participants were either US-born or immigrants. They could belong to any race/ethnicity. We also included samples from all years between 1972 and 2021.

### 2.3. Measures

#### 2.3.1. Predictor

*Education*: Years of schooling were self-reported and measured as education. This was a continuous variable.

#### 2.3.2. Dependent Variable

*Poor Self-Rated Health (SRH).* The outcome was poor SRH, measured by a single item measure asking a question from the respondents about, “How do you rate/describe your health overall?”. We considered 1 for fair or poor health and 0 for good and excellent health.

#### 2.3.3. Moderator

*Immigration.* Immigration status was the moderator variable determined through the following question: “Were you born in the US?”. “No” answers, equivalent to immigrant status, were recorded as 1, and “Yes” answers equal to native-born participants were coded as 0.

#### 2.3.4. Confounders

In this study, age, sex, survey year, and race/ethnicity were considered confounders. Age (years) was a continuous variable ranging from 18 to 90, and sex was a dichotomous variable, coded 1 for men and 0 for women. Race was a three-level nominal variable: Black, White, and other. Race was self-identified by the individual. For race, White was considered the reference group. Survey year was a number ranging from 1972 to 2021. This variable was treated as a continuous measure.

### 2.4. Statistical Analysis

Statistical Package for Social Sciences (SPSS) version 24.0 (IBM Inc., Armonk, NY, USA) was used for our data analyses. Descriptive statistics were reported as frequency (%) and mean (±standard deviation) for categorical/nominal and numerical variables, respectively. For bivariate analysis, we used independent samples t-test and Chi-square to compare immigrant and non-immigrant older adults. For multivariable analysis, four multivariable logistic regression models with poor SRH as the primary outcome were used. In all these models, age, sex, survey year, and race/ethnicity were covariates. *Model 1* and *Model 2* were performed in the pooled sample. *Model 1* did not include any interaction term; however, *Model 2* included immigration status by education as an interaction term. *Model 3* and *Model 4* were specified in the US-born participants and immigrants, respectively. We tested our central hypothesis in *Model 2* with the significance of the interaction term between immigration status and education. This approach tests population differences in correlates of factors, such as education or poor SRH. The reason we still performed *Model 3* and *Model 4* is that covariates may have different roles across groups. To observe any group difference in the role of covariates, we performed the final two models. The logistic regression models reported adjusted unstandardized regression coefficients (b) and corresponding 95% confidence intervals (CIs) and *p* values.

## 3. Results

### 3.1. Descriptive Data

Overall, 7999 older adults entered our analysis. These participants were sampled between 1972 and 2021. Table 1 summarizes the demographic characteristics of all participants based on immigration status. Bivariate tests showed that immigrant and US born were different in terms of gender, SRH, race, age, and education. Compared to US-born people, immigrants had lower education, they were older, and less likely to be White and female (*p* < 0.05 for all comparisons).

### 3.2. Logistic Regression in the Overall Sample

Table 2 summarizes the results of two logistic regressions in the pooled sample of older adults, with poor/fair SRH as the primary outcome, educational attainment as the predictor, and age, sex, survey year, and race as covariates. *Model 1*, which did not include any interaction term, showed the main effect of education. According to the information revealed by *Model 1* in the pooled sample, higher education was correlated with lower odds of poor/fair SRH (main effect OR = 0.871, CI = 0.858–0.884, 95% *p* = 0.000).

*Model 2* demonstrated an interaction between education and immigration status on the reported poor/fair SRH (interaction OR = 1.066, CI = 1.023–1.110, *p* = 0.003). Therefore, the protective effect of education against poor/fair SRH is more robust in US-born participants than in their immigrant counterparts.

### 3.3. Stratified Logistic Regressions

Table 3 shows the logistic regression results in US-born participants (*Model 3*) and the logistic regression in immigrants (*Model 4*). These *models* show that the effect of higher education against poor/fair SRH was more prominent for US-born (OR = 0.865, 95% CI = 0.851–0.879, *p* = 0.000) than older immigrant (OR = 0.919, 95% CI = 0.881–0.959, *p* = 0.000) adults. These models also showed that covariates operate differently for our two groups. While survey year was correlated with SRH in US-born older adults, this association was not significant for immigrant older adults.

## 4. Discussion

Although previous studies have made significant contributions to our understanding of diminished health returns of education among marginalized groups, there is a need to examine whether this pattern extends to older adult immigrants in the US. Building upon previous work, this analysis included data from 7999 US older adults (aged 65+) who participated in the GSS from 1972 and 2021. Our findings revealed that as education increases, SRH improves. This is in line with established research of the effect of socioeconomic status on health. In addition, we found that US-born older adults benefited more from their education in terms of SRH compared to immigrant older adults. In other words, education’s heath effect was weaker for immigrants than US-born older adults. This study is the only study, according to our knowledge, on the moderating effect of immigration status on health returns of education in older adults in the US.

This study extends the literature of health returns of education to older adults in the US. Other studies have considered the moderating effect of immigration status on the health effects of education and income in adults and young children. One study in the US showed the protective effects of higher education on decreasing the risks of psychological distress and chronic diseases and improving subjective health status are weaker for immigrant than US-born populations [29]. Another study investigated the MDRs of household income on children’s depression as a function of immigration using cross-sectional data of 6412 children between the ages of 9–10-year-old in the United States. The study compared non-immigrant and immigrant children and examined the effect of household income on children’s depressive symptoms. Overall, high household income was associated with lower depressive symptoms among children. However, immigration status showed a statistically significant interaction with household income on children’s depressive symptoms. The significant interaction suggested that high household income had a smaller protective effects against depression for immigrant than non-immigrant children [51]. Similarly, another study in young adults finds that high education and income were associated with lower odds of current cigarette smoking, and that immigration showed significant statistical interactions with both education and income. These significant interaction terms were suggestive of weaker protective effects of high education and income on reducing prevalence of current cigarette smoking for immigrant than non-immigrant adults [52].

Why might older adult immigrants experience diminished health returns with higher levels of education compared to US-born counterparts? One of the mechanisms for the protective effects of education is that high education is associated with lower levels of various types of stress, including but not limited to discrimination; however, this association may differ across majority and minority populations [53]. Immigration status may reflect personal experiences, including an individual’s cultural background and perceived marginalization [29]. Repeated exposure to discrimination is often cited as a reason for stress and poor health among immigrant groups. Additionally, the act of migration itself is a stressful process with negative psychosocial consequences on as immigrants become integrated into the US society. A survey of Turkish and Polish immigrants in Germany showed that the frequency of reporting discrimination among higher education participants depends on the “bright boundaries” [54] they faced. Immigrants who experience more bright boundaries may face additional difficulties in integrating into the host country. In that study, for example, Turkish immigrants experienced more discrimination and had more difficulties with assimilation as they thought of themselves as a different community [55]. On the other hand, immigrants may be motivated to pursue higher education. Thus, the association observed in cross-sectional studies may have unexpected consequences longitudinally [56].

There is a need to better understand broader social factors and determinants of SRH and disparities in highly educated immigrant populations. However, immigration status as a variable may not be enough to capture more nuanced experiences, including level of acculturation, citizenship status, generation status, or even context of migration (like forced displacement). In the post-migration context, the neighborhood environment, like ethnic density or ethnic enclaves and sanctuary cities, may alter the protective effects of education for immigrants and may be a direction for future research. Additionally, complex associations may be observed for SRH across subgroups of immigrants. For example, skin color and accent may explain why some groups of immigrants’ experience discrimination. Data analysis from 1800 Black and White participants showed a direct positive association between education and SRH [57]. However, these associations were more prevalent among Black participants than White Americans [57]. Another study based on the data of 2606 Europeans showed that discrimination reduces SRH among first-generation immigrants from low-income countries who live in European countries but not among their descendants [58]. All these studies indicate a great amount of complexity in the experiences of immigrants and the implication of such complexities for SRH within and across immigrant groups. Additionally, there is a need to examine mechanisms that may reduce the health returns of education for older adult immigrants [25]. Navigating an English-speaking environment can pose unique challenges for older adult immigrants, as they experience social isolation or barriers to healthcare due to restrictive immigration policies. Restrictive immigration policies are also associated with higher stress levels, stigma, and discrimination among immigrants [59].

## 5. Limitations

This was a cross-sectional study of US older adults with a few limitations. Our study had a few older immigrant adults, which may have implications for study power. Good SRH may also be a driver for pursuing higher education [56], thus inferring any causal association between education and poor SRH is impossible. Moreover, our data did not include any information regarding immigrants’ length of stay. It is shown that new immigrant responders report good SRH more frequently [55]. Additionally, we analyzed data from various states and time intervals with different policies regarding immigrants. We need to study how much immigrants feel assimilated into the newly adopted culture [55]. Furthermore, no information was available regarding the country where the individuals had received their education. According to Zhang et al., immigrants who had completed their education outside the US reported less discrimination than those who studied in the US [60]. Further studies should evaluate if there are any differences between US-born and immigrants regarding the positive impact of education on health outcomes.

## 6. Conclusions

This study found that education is protective against poor SRH among US older adults; however, this effect is more robust in US-born than immigrant older individuals. Future studies are needed to evaluate the mechanisms that reduce the health returns of education on various health outcomes in immigrants. Policy solutions for the elimination of health disparities in immigrant populations require interventions that address barriers that hinder highly educated immigrants as well. Such policies should reduce anti-immigrant discrimination, for example. Researchers should also not assume that immigration only has a main effect on health as some of its effect is through modifying the effects of education on health.

## Figures and Tables

**Table 1:** Distribution of demographic factors overall and by immigration status.

	All *n* = 7999		US-Born *n* = 7473		Immigrant *n* = 526	
	Frequency	%	Frequency	%	Frequency	%
Gender (%) *						
Women	4806	60.1	4524	60.5	282	53.6
Men	3193	39.9	2949	39.5	244	46.4
Self-Rated Health (SRH) *						
Excellent-Good	4970	62.1	4663	62.4	307	58.4
Poor/Fair	3029	37.9	2810	37.6	219	41.6
Race *						
White	6926	86.6	6522	87.3	404	76.8
Black	850	10.6	811	10.9	39	7.4
Other	223	2.8	140	1.9	83	15.8
	Mean	SD	Mean	SD	Mean	SD
Age * (Mean, SD) (65–89)	73.85	6.624	73.81	6.619	74.46	6.669
Education (0–20) *	12.17	3.688	12.22	3.595	11.48	4.768

* *p* < 0.05 for comparison of immigrant and US-born groups.

**Table 2:** Pooled sample logistic regression models.

	B	S.E.	Exp(B)	95% C.I. for EXP(B)		Sig.
**Model 1 (All, Main Effects)**						
Immigrant	0.011	0.100	1.011	0.831	1.228	0.916
Race						
White						
Black	0.450	0.078	1.568	1.346	1.827	0.000
Other	0.464	0.149	1.590	1.188	2.129	0.002
Survey year	−0.007	0.002	0.993	0.989	0.997	0.000
Age	0.021	0.004	1.021	1.014	1.029	0.000
Gender (Male)	0.039	0.050	1.040	0.944	1.146	0.430
Education (Years)	−0.138	0.008	0.871	0.858	0.884	0.000
**Model 2 (Model 1 + Interactions)**						
Immigrant	−0.702	0.256	0.496	0.300	0.818	0.006
Race						
White						
Black	0.437	0.078	1.549	1.329	1.805	0.000
Other	0.431	0.148	1.539	1.152	2.056	0.004
Survey year	−0.007	0.002	0.993	0.989	0.997	0.001
Age	0.021	0.004	1.022	1.014	1.029	0.000
Gender (Male)	0.042	0.050	1.043	0.946	1.149	0.400
Education (Years)	−0.146	0.008	0.864	0.850	0.878	0.000
Immigrant × Education (Years)	0.064	0.021	1.066	1.023	1.110	0.003

**Table 3:** Stratified logistic regression models.

	B	S.E.	Exp(B)	95% C.I. for EXP(B)		Sig.
**Model 3 (US-Born)**						
Race						
White	ref					
Black	0.468	0.080	1.597	1.365	1.869	0.000
Other	0.386	0.182	1.471	1.030	2.101	0.034
Survey year	−0.007	0.002	0.993	0.989	0.997	0.001
Age	0.019	0.004	1.020	1.012	1.027	0.000
Gender (Male)	0.057	0.052	1.059	0.957	1.171	0.268
Education (Years)	−0.145	0.008	0.865	0.851	0.879	0.000
**Model 4 (Immigrant)**						
Race						
White	ref					
Black	−0.200	0.368	0.819	0.398	1.684	0.587
Other	0.443	0.268	1.558	0.921	2.634	0.098
Survey year	−0.001	0.007	0.999	0.985	1.014	0.920
Age	0.048	0.014	1.049	1.021	1.079	0.001
Gender (Male)	−0.130	0.185	0.879	0.611	1.263	0.484
Education (Years)	−0.084	0.022	0.919	0.881	0.959	0.000

## Data Availability

Data of the GSS are available here: https://gss.norc.org/Get-The-Data (accessed on 12 January 2022).
